# Celebrating the first 20 years of publication of Primary Health Care Research & Development!

**DOI:** 10.1017/S1463423618000956

**Published:** 2019-01-23

**Authors:** Sally Kendall, Ros Bryar, Katie Henderson

**Affiliations:** 1 Centre for Health Services Studies, University of Kent, Canterbury, UK; 2 Cambridge University Press, Cambridge, UK

The publication of the first edition of Primary Health Care Research & Development (PHCR&D) in January 2000 was planned to coincide with the launch of the United Nations Millennium Development Goals (MDG) (http://www.un.org/millenniumgoals/) and the culmination of the Health for All by the Year 2000 strategy (https://www.who.int/whr/1998/media_centre/executive_summary6/en/), both significant milestones for researchers and practitioners in primary health care. Since 2000 there have been further significant developments in primary health care and in the history of the Journal. In celebration of the 20th anniversary of PHCR&D the Editors in Chief would like to record, through this Editorial, some of these developments that track a journal from small beginnings to one that now has international standing in the primary health care academic and practice communities.

When PHCR&D was envisioned in 1998 the 20th anniversary of the Declaration of Alma Ata [World Health Organisation (WHO), 1978] was being celebrated. The global health community was still looking towards the millennium, and Health for All in the year 2000 was the goal that primary health care was going to be pivotal in achieving. Research in primary care was developing rapidly in 1998 with growth in the United Kingdom in academic departments of general practice and a new interest in Primary Care Research Networks and research stimulated by the Mant Report (1997).

This fledgling interest in discovering what works at a local level in primary care stimulated the emergence of a new international, academic journal that would focus not only on research but also on development (the application of research to practice). The aim was that the Journal would be attractive not only to academics in primary care but also to practitioners, GPs, nurses, midwives, dieticians, pharmacists – indeed all those engaged in research and development work that impacts on individuals, families and communities before or after they require hospital care. This unique combination of research and development enabled the launch of PHCR&D in 2000 at a moment when there was an appetite for dissemination of both of these types of studies. We published Editorials at that time both to define primary health care (Bryar, 2000) and to emphasise and promote the importance of research and development to the primary care community (Kendall, 2000), and it soon became apparent that there was indeed room in the field for a new journal with an extended scope.

In 2008 the WHO published the report: ‘Primary Health Care Now More than Ever’ (WHO, 2008). This was an important moment for PHCR&D as the report re-emphasised the central role of primary care, the importance of collaboration and the value of a public health/population perspective in primary care, all themes addressed in papers in the Journal over the years. Another milestone for the Journal and for primary health care was the publication in 2015 of the 17 United Nations (2015) Sustainable Development Goals (SDGs), all of which impact on the health and well-being of individuals, communities and populations. While the MDGs may have been perceived as having greater relevance for less developed countries, the SDGs have world-wide relevance (https://sustainabledevelopment.un.org/?menu=1300) and help to provide a framework for research and development activity in primary health care. Then in 2018 came another important endorsement of the role of high quality primary health care with the WHO launch of the Declaration of Astana (WHO, 2018) during the celebrations of the 40th Anniversary since the Declaration of Alma Ata. The new Declaration was signed by WHO member states in Astana, Kazakhstan and stated:

*We are convinced that strengthening primary health care (PHC) is the most inclusive, effective and efficient approach to enhance people’s physical and mental health, as well as social well-being, and that PHC is a cornerstone of a sustainable health system for universal health coverage (UHC) and health-related Sustainable Development Goals.*

(WHO, 2018)

Primary health care is thus central to achieving the SDGs globally and providing Universal Health Coverage (UHC), all of which requires strong evidence from research and practice. In support of this PHCR&D is delighted to be publishing a special collection in 2019 that celebrates 40 years since Alma Ata and the future contribution of PHC. The Editorial authored by colleagues from WHO in September 2018 (Kluge *et al*., 2018) heralded this issue and drew attention to the importance of UHC in times of global uncertainty and suffering.

Over these years PHCR&D has moved through a number of transitions and partnerships. The Journal was initially published by Arnold Publishers which was part of Hodder Education and we are for ever grateful for the support of staff at Arnold for believing in this new journal. In 2006, Arnold disinvested in journals and PHCR&D was fortunate to move to Cambridge University Press, a publisher which has nurtured and supported the Journal ever since. We are very grateful to all the staff at the Press who have supported the Journal but in particular to Dan Edwards, Publisher and Katie Henderson, Editor, whose wisdom and calm advice has kept us on track since 2006. We are very pleased that every paper ever published in PHCR&D is archived by the Press and searchable on the website https://www.cambridge.org/core/journals/primary-health-care-research-and-development/all-issues.

The Journal has been fortunate in these 20 years to have had two partnerships with academic membership organisations. The first was with the UK Society for Academic Primary Care (SAPC; https://sapc.ac.uk/). The SAPC provided us with an engaging community and many members submitted papers to PHCR&D. The support of key leaders in SAPC including Professor Helen Lester, Professor Debbie Sharp and Professor Amanda Howe was enormously valuable to the development of the Journal and work towards achieving an initial Impact Factor.

More recently in 2016, we became the official journal of the European Forum for Primary Care (EFPC; http://www.euprimarycare.org/), thereby reinforcing our position as an internationally relevant journal. It has been widely promoted at the EFPC conferences and other events and the Editors have held workshops at EFPC conferences to stimulate writing and reviewing for the Journal.

Another partnership which the Journal has established is with the Cochrane Collaboration and this has led to publication of Cochrane summary papers enabling readers to receive short digests of Cochrane reviews on a range of PHC-related topics. These have received a strong citation rate indicating that researchers are making use of the summaries in their own research and publications with, hopefully, a wider impact on the quality of primary care.

Citation rates have been critical to the work that the Journal and staff at the Press have supported which led to PHCR&D in 2016 receiving an initial impact factor, a milestone in the history of the Journal. The following year the Journal gained an Impact Factor of 1.12 ranking it 16th out of the 20 international journals in the field of primary care.

There is much to celebrate in 20 years of PHCR&D, but the Editors could not have achieved it without the support of our fantastic Associate Editors, Editorial Board Members, wonderful peer reviewers, people who write papers for the Journal, and Valerie Dennis who manages the online system Editorial Manager and keeps all of us on track. We thank you all.

Sadly, we have also lost some great colleagues and supporters over the past 20 years and we would particularly like to remember:

Dr Lisbeth Hockey

Muriel Lee

Professor Yvonne Carter

Professor Helen Lester

They were giants in PHC, we think of them with warmth and gratitude for their contributions, support and faith in PHCR&D.

At the same time as research and development in PHC has been expanding and changing, so has the format and access to PHCR&D changed over the years. From publishing in print four times a year in 2000 the Journal moved to print and online publication and then to online publication six times a year and, now 2019 heralds a new era for PHCR&D, as it moves to continuous publication where articles will be published in the volume as and when they are accepted.

We are also excited to announce that from 1 January 2019 onwards, PHCR&D will be moving to a wholly Gold Open Access model of publication. This means that articles (upon payment of an article processing fee) will be made freely available in perpetuity to all. Articles will be published under a Creative Commons Attribution License (CC-BY), which allows the use, distribution, reproduction and adaptation of an article in any medium, provided that the original work is properly cited. It also permits anyone, regardless of where they are in the world, to read, distribute and cite papers published in PHCR&D, helping to increase visibility, dissemination and usage of key research published in the field.

This move comes in response to a growing global need driven by authors, institutions and funders to move towards a more open future in the world of scholarly publishing. There have been a diverse range of reactions from academic publishers in response to this need, and a continued discussion at every level of the publishing infrastructure regarding how best to ensure that our publishing is both sustainable and accessible.

These developments are not exclusive to Europe alone: in June of this year, the University of California’s Systemwide Library and Scholarly Information Advisory Committee (SLASIAC) issued a Call to Action (Office of Scholarly Communication, University of California, 2019): ‘We believe the time has come to address these issues head-on through a combined strategy that places the need to reduce the University’s expenditures for academic journal subscriptions in the service of the larger goal of transforming journal publishing to open access. Through our renewal negotiations with publishers, we will pursue this goal along two complementary paths: by reducing our subscription expenditures, and investing in open access support’.

Funding bodies are also playing an increasingly important role in the Open Access movement. In September of this year a group of 11 organisations (including the European Commission) formed a group known as cOAlition S, which has since grown to (at the time of writing) 14 members and now includes the Wellcome Trust and the Gates Foundation amongst its members. Together, cOAlition S has unveiled Plan S – a 10-point policy which aims to accelerate the transition to a fully Open Access publishing landscape (cOAlition S, 2018a). Signatories of this plan have committed to 10 core principles, which aim to achieve that all research coming out of public grants provided by participating members must be published in a fully Gold Open Access journal (or on an OA compliant platform) by 2020. Plan S has since been amended to allow a transition period for ‘hybrid’ journals, which publish both subscription and Open Access articles (cOAlition S, 2018b).

Whilst Plan S is only a very recent (and changing) development, it is clear to see that it, in addition to a general increase in the amount of Open Access articles published in some Cambridge titles, is part of a broader move towards a more open world in the field of scholarly publishing, as institutions move away from paying for subscriptions and towards supporting Open Access publication (Piwowar, 2018).

As a result of these recent developments and the growth of Gold Open Access content in PHCR&D, we decided to ‘flip’ the Journal from a subscription model to a wholly Open Access one. Our primary focus is to support our community by continuing to provide the highest quality research and to support our authors, allowing them to comply with mandates from their university and their funders, and also increasing the dissemination of their research for the benefit of all.

While there are many exciting benefits to this move to full Open Access, we do recognise that it does not come without challenges. We are fully aware that there are a range of differing levels of funding available in each sub-discipline, and that some among our PHCR&D authors may at present have limited access to funding for Open Access publishing. We want PHCR&D to remain an inclusive environment for the whole community and to that end we offer a generous waiver policy, with some article types exempt from an Open Access article processing charge entirely. Please see our Instructions for Contributors for details of this policy.

We hope you are as excited by this change as we are. We have some fantastic content coming up for PHCR&D in 2019, including the special collection on ‘40 years since the Alma Ata declaration’. Under the new wholly Gold Open Access model, we are pleased to be able to share this to the benefit of all, ensuring that the Journal has both an exciting and sustainable future ahead.
